# A model of urban scaling laws based on distance dependent interactions

**DOI:** 10.1098/rsos.160926

**Published:** 2017-03-22

**Authors:** Fabiano L. Ribeiro, Joao Meirelles, Fernando F. Ferreira, Camilo Rodrigues Neto

**Affiliations:** 1 Departamento de Física (DFI), Universidade Federal de Lavras (UFLA), Caixa Postal 3037, 37200-000 Lavras, Minas Gerais, Brazil; 2 Laboratory on Human-Environment Relations in Urban Systems—HERUS, École polytechnique fédérale de Lausanne (EPFL) Station 2, 1015 Lausanne, Switzerland; 3 EACH—Universidade de São Paulo (USP), Av. Arlindo Bettio, 1000 (Vila Guaraciaba), 03828-000 São Paulo, SP, Brazil

**Keywords:** agglomeration effects, complex systems, power laws, scaling laws, urban indicators, public policies

## Abstract

Socio-economic related properties of a city grow faster than a linear relationship with the population, in a log–log plot, the so-called *superlinear scaling*. Conversely, the larger a city, the more efficient it is in the use of its infrastructure, leading to a *sublinear scaling* on these variables. In this work, we addressed a simple explanation for those scaling laws in cities based on the interaction range between the citizens and on the fractal properties of the cities. To this purpose, we introduced a measure of social potential which captured the influence of social interaction on the economic performance and the benefits of amenities in the case of infrastructure offered by the city. We assumed that the population density depends on the fractal dimension and on the distance-dependent interactions between individuals. The model suggests that when the city interacts as a whole, and not just as a set of isolated parts, there is improvement of the socio-economic indicators. Moreover, the bigger the interaction range between citizens and amenities, the bigger the improvement of the socio-economic indicators and the lower the infrastructure costs of the city. We addressed how public policies could take advantage of these properties to improve cities development, minimizing negative effects. Furthermore, the model predicts that the sum of the scaling exponents of social-economic and infrastructure variables are 2, as observed in the literature. Simulations with an agent-based model are confronted with the theoretical approach and they are compatible with the empirical evidences.

## 1. Introduction

When humanity built the first cities, they brought together individuals previously separated by space, increasing social and economic interactions, through the shared infrastructure of the cities, in a more constant and efficient way. Since then, the city as a social organization of humanity, has become an important place to create interactions in space and time between individuals [1].

Following Warren Weaver’s ideas on organized complexity [2], Jane Jacobs first proposed the idea of the city as an integrator of individuals in her seminal work, *The Death and Life of the Great American City* [3]. In her work, Jacobs saw cities as complex entities, organized in a bottom-up manner, by the behaviour of its inhabitants. In the book’s concluding chapter, Jacobs defines the city as a problem of organized complexity. The ideas of complexity were young then, and Jacobs quickly realized the importance of understanding cities from this conceptual framework. Thereafter, this complexity approach to cities have yielded some important insights into urban science [4–7].

In the same vein, the hypothesis raised by Bettencourt & Lobo [8] proposes a new explanation based fundamentally on network effects: the mere fact that individuals are spatially close to each other increases the number of potential encounters between them. Theoretically, the probability of encounters and interaction grows as the individuals get closer and this process lowers the transaction and communication costs.

Recent research made it possible to empirically support these propositions [1,9,10], thanks to the increasing availability of urban data, from official statistics to private databases. Taking advantage of this large volume of data, covering thousands of cities in the world, researchers have apparently found statistical regularities on how concentrations of people affect economic activities, infrastructure and social vitality [11].

The main findings from this new field of study, integrating urban planning, geography and physics of complex systems, can be summarized by the assumption that all cities produce spatial scale economies as they grow and at the same time, achieve gains in their socio-economic productivity. In general, when different cities within the same urban system (i.e. in the same country) are compared, the largest cities are denser than the smallest towns, and therefore the total amount of infrastructure per inhabitant is smaller. At the same time, as a general rule, the largest city is richer, costlier and more culturally and technologically productive when the metrics *per capita* are analysed.

If the socio-economic variables of a city grow faster than the population, we have an effect called *superlinear scaling* [10], characterized by a scaling exponent greater than one. The data indicate that the urban infrastructure follows a similar law: the larger a city, the more efficient it is in the use of its infrastructure, leading to significant economies of space. For example, when the population of a city doubles its material infrastructure grows less than that, from the number of gas stations to the total length of water pipes, streets and electric cables [9]. If the infrastructure grows less than the population, we have something called *sublinear scaling*, characterized by a scaling exponent smaller than one. Last, there is a third class of variables which presents *linear scaling* with the population size. According to empirical evidence, those variables are related to individual needs, as consumption of water and electrical energy, number of employment, and so on [9].

The most remarkable properties of the urban scaling hypothesis are (i) the concentration of people in space and time and (ii) greater intensive use of urban infrastructure. Together, (i) and (ii) promote matching and social coordination, increasing social indicators such as wealth, innovation and crime, enabling better use of infrastructure such as the street and transportation networks, electrical and communication cables and many others as a city grows [10]. Some evidence, however, indicates a considerable sensitivity of the scaling exponent in relation to the adopted definition of city, challenging the universality hypothesis of these scaling laws [12–15]. More empirical evidence is clearly needed in this discussion but theoretical understanding, as presented in this work, can contribute to this dispute.

In order to build a quantitative theory of cities, we must take into account the city’s geometry to calculate aggregate amounts that generate social and infrastructure indicators. According to the statistical mechanics approach, to explain the scaling law observed in many complex systems we can ignore several microscopic details and focus on the important features. More specifically, we can focus on proposing a spatial interaction model involving the individuals and how they make choices about their destinations or how they are influenced by the social network.

In the work herein, we look for a simple explanation for the scaling laws in cities based on the fractal properties of the cities, as well as the behaviour of individuals. For that, we introduce a measure of social potential that captures the influence of social interaction on the economic performance and the benefits of amenities in the case of infrastructure offered by the city. We assume that the populational density depends on the fractal properties and that the individual interaction intensity decays with distance. As a result we obtain a power law scaling for social indicators and infrastructure. The scaling exponents we found are coherent with the empirical data. An agent-based model is proposed to compute experiments and to test the hypotheses.

The paper is organized in five sections, including this introduction. Section 2 presents the model based on the hypothesis just exposed above. Section 3 presents the model simulation results and §4 discusses our results. We conclude in §5 with suggestions of further research.

## 2. The model

### 2.1. Scaling laws in the cities: socio-economic production

Individuals with limited information can take advantage from another person’s opinions, behaviours and abilities in order to fulfil their needs and solve their problems. Through a positive feedback mechanism, those interactions allow individuals to explore the information and resources available in the city to enhance creativity and socio-economic production. Each individual in the city is stimulated by the others. In this context, we use the word *stimulus* to mean all the social characteristics that influence the individual, such as cultural attitude, dialect and demand of products, just to cite a few.

The distance between individuals in a city is an important factor. For instance, the chance of two individuals meeting each other through common friends depends on the distance they live one from each other. Moreover, the impact or influence which one person has on another also may depend on the distance between them. Based on this, let us consider the stimulus strength of the individual *j* over the individual *i* be represented by the function *f*(*r*_*ij*_), where *r*_*ij*_ is the distance between them. So, based on recent empirical evidence [16,17], it is quite plausible to assume that the stimulus between two citizens decays with the distance, according to:

2.1 $$f(r)=\left\{ \begin{array}{ll} \frac{1}{r^{\gamma }} & \text{if }r>2r_{0}\\ \frac{1}{{\left(2r_{0}\right)}^{\gamma }} & \text{otherwise}, \end{array}\right.$$

where *γ* is the decay exponent. This function was first used to model the interaction of living cells in a competitive [18] and cooperative [19–21] environment, and recently it was used to model the interaction between tumour cells [22]. The distance 2*r*_0_ is the minimal distance between the agents (the distance of a house, for instance). For convenience purposes, we assume that $r_{0}=\frac{1}{2}$. We find empirical support for the hypothesis expressed by equation (2.1), with experimental values of *γ* ranging in the interval: 1≤*γ*≤1.5 [17]. For example, the distribution of physical distances of Facebook contacts are *γ*=1.03 [16] and *γ*=1.12 [23]; the distribution of e-mail distances: *γ*=1.0 [16] and *γ*=1.20 [23]; and the frequency of cell phone call with the distance is found in the interval: 1≤*γ*≤1.5 [17].

The influence between individuals separated by longer distances may be small, but if many people share the same opinions, their collective strength may be meaningful and affect them. The extent of the social tissue, measured by the size of the social network, contributed to the amount of social stimulus or influence to which one individual is exposed. For simplicity, we proposed an additive model to measure the total effective influence or stimulus. The individual stimulus of a single individual *i* from his/her interaction with all the other (*N*) citizens of the city (influences), can be written as

2.2 $$I_{i}=\sum_{j=1}^{N}f(r_{ij}),$$

while the socio-economic stimulus of the city is given by

2.3 $$I_{\mathrm{se}}=\sum_{i=1}^{N}I_{i}.$$

We are not modelling the mechanism behind how an individual may influence others. We are just considering that the influence strength decays over the social network through distance in a pairwise interaction framework at the micro level. Moreover, we are hypothesizing that the aggregate information emerging from individual interaction is the mechanism for the power law observed at the macro level, as presented in the following.

Considering that the individuals are continually distributed along the city area with density *ρ*(**r**), where **r** is a position vector, then the sum in the equation (2.2) can be approximated by an integral. That is, the total stimulus felt by *i* can be computed by

**Figure 1.**
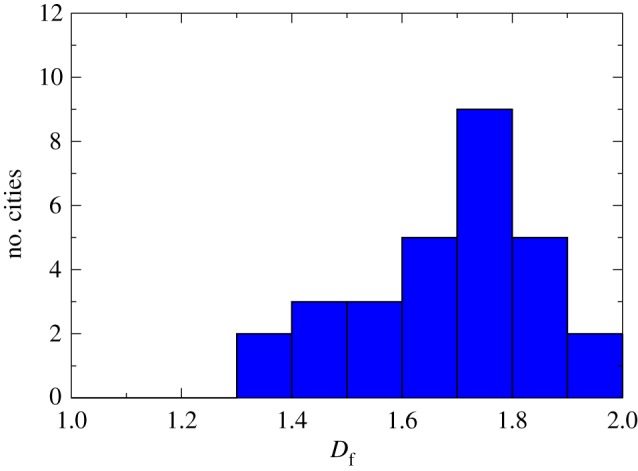
Histogram of the fractal dimension of many cities around the world. Data collected from [24], p. 242. Most of the cities studied have their fractal dimension in the interval *D*_*f*_=[1.6,1.8], with average ${\bar{D}}_{f}=1.7$.

2.4 $$I_{i}=\int_{\mathrm{all}\;\mathrm{space}}\rho (\text{r})f(r)d^{D}\text{r},$$

where *D* is the Euclidean dimension of the space in which the population is embedded. In the case of a city, which lies in a plane, we can assume that *D*=2. However, following the approach adopted in many studies, the spatial distribution of a city obeys fractal behaviours [24,25]. Figure 1 presents a histogram built from a table of the fractal dimension *D*_*f*_ of many cities around the world presented in [24], p. 242. The *box-counting method* was used to compute the fractal dimension of the cities [26,27]. According to the data, most of the cities have their fractal dimension in the interval *D*_*f*_=[1.6,1.8], with average ${\bar{D}}_{f}=1.7$, as shown in figure 1. There are other references that suggest this is the average value of the fractal dimension of the cities [28].

Cities usually present fractal behaviour, thus we assume that *r* is the linear length, and then the number of individuals must scale as *r*^*D*_f_^, while the area that embedded the population scales as *r*^*D*^. Thus, we write the population density equation as

2.5 $$\rho (\text{r})=\frac{\text{number of individuals}}{\text{Area}}=\rho_{0}\frac{r^{D_{f}}}{r^{D}}=\rho_{0}r^{D_{f}-D},$$

where *ρ*_0_ is constant (for details, see [27]). This hypothesis suggests that the density is a radially symmetric function. With this hypothesis, we solve equation (2.4) using

2.6 $$d^{D}\text{r}=r^{D-1}\;dr\;d\Omega_{D}$$

(transforming from Cartesian coordinates to hyperspherical coordinates). Using the periodic boundary condition, we have

2.7 $$I_{i}(N)=\frac{\omega_{D}}{D_{f}}\frac{1}{1-\gamma /D_{f}}\left[{\left(\frac{\omega_{D}}{D_{f}}N\right)}^{1-\gamma /D_{f}}-1\right]+\frac{\omega_{D}}{D_{f}},$$

where *ω*_*D*_ depends only on the Euclidean dimension (for details, see [19–21]). The right side of the above equation does not depend on the index *i*, which means that, given the hypothesis presented, all the individuals of the population felt the same stimulus intensity, which depends on the population size of the city. It is due to the self-similarity of the fractal structure formed by the population [27]. In this way, the individual stimulus scales with *N* by

2.8 $$I_{i}(N)=c_{1}N^{1-\gamma /D_{f}}+c_{2},$$

where *c*_1_ and *c*_2_ are constants. The ratio *γ*/*D*_f_ plays an important role in this context. In fact, this ratio is responsible for the range of interaction between each pair of individuals in the city. If (*γ*/*D*_f_)>1, the individual stimulus given by (2.4) does not depend on the population size (given a population sufficiently large). Let us call this situation the *short-range interaction regime* since the individual interacts only with its closer neighbours. In this case, the stimulus felt by a single individual converges to a constant when the population size is large enough. On the other hand, if (*γ*/*D*_f_)<1, the individual stimulus given by (2.4) diverges when N→∞ as a consequence of the fact that the individuals interact effectively with everybody, despite being less intense with people who are further away. We will call this case the *long-range interaction regime*.

From equation (2.4), the total socio-economic stimulus produced in the city, that is *I*_se_=*NI*_*i*_, is given by

2.9 $$I_{\mathrm{se}}=c_{1}N^{\beta_{\mathrm{se}}}+c_{2}N$$

where

2.10 $$\beta_{\mathrm{se}}\equiv 2-\frac{\gamma }{D_{f}},$$

is the scaling exponent associated with the socio-economic activities. So the total stimulus in the city is a power law of the population size.

In the short-range interaction regime, *γ*/*D*_f_>1, the total stimulus of the city scales as *I*_se_∼*N* (linear behaviour), which is not compatible with the empirical data presented in table 1. It means that if the individuals of the city interact only with their neighbours and not with the distant ones, the intellectual and socio-economic activities of the city will not scale superlinearly, but only linearly with the population size. Conversely, in the long-range interaction regime, that is *γ*/*D*_f_<1, the total stimulus scales as

2.11 $$I_{\mathrm{se}}∼N^{\beta_{\mathrm{se}}},$$

with *β*_se_>1. This result agrees qualitatively with the empirical data presented in table 1.

**Table 1.** Empirical results of the scaling exponent for two particular indicators of the cities in three different countries: one correlated to an infrastructure indicator (gas station) and other related to a socio-economic one (GDP). The data came from [8,29], but similar results were presented in other studies [9,10,29]. The number of gas stations scales sublinearly ($\beta \approx \frac{5}{6}<1$), while the GDP scales superlinearly ($\beta \approx \frac{7}{6}>1$) with the population size of the cities. Different countries present similar scale exponent values, and the evidence suggests a correlation between the exponents, given that the sum, no matter the country, is around 2.

country	*β*_infra_ (gas stations)	*β*_se_ (GDP)	*β*_infra_+*β*_se_
France	0.90 [0.8, 1.0]	1.20 [1.15, 1.26]	2.1 [1.95, 2.2]
Spain	0.75 [0.65, 0.85]	1.13 [0.97, 1.30]	1.88 [1.62, 2.15]
Germany	0.80 [0.75, 0.85]	1.17 [1.06, 1.28]	1.97 [1.81, 2.13]

This means that, according to the theory presented here, if a city presents superlinear behaviour of their socio-economic indicators, then the individuals (or the regions) of this city must be able to interact with all other individuals (or other regions) of this city. In other words, the city will only enhance their socio-economic indicators if the city behaves as a whole. On the contrary, that is, if the city is just a collection of isolated regions, then the socio-economic indicators will be only linearly dependent on the population size. As we presented, the last case is not supported by empirical data.

Medellin, Colombia, is a example of such a situation. This city had a strong increase in development indicators when it began to integrate its less developed areas, previously isolated in the hills, with the rest of the city. This integration process, made by the implementation of the aerial lift (or metrocable), generated new revenue for the inhabitants of these places and promoted the development of the city as a whole [30].

### 2.2. Scaling laws in the cities: infrastructure

**Figure 2.**
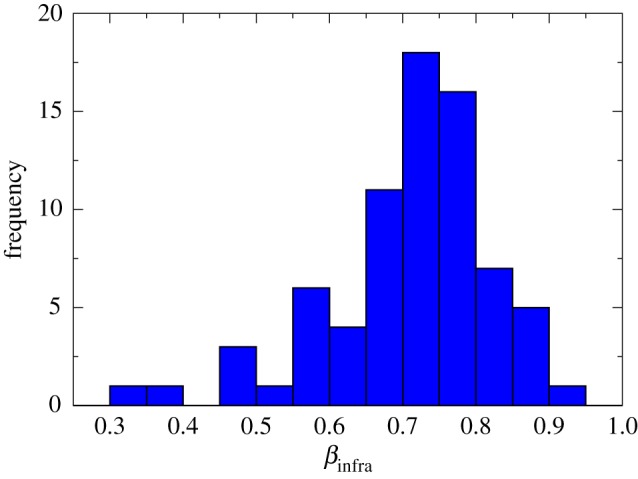
Histogram showing the number of amenities with a particular value of scaling exponent (*β*_infra_). This histogram was built with 74 amenities (for instance, bakery (*β*_infra_=0.847), beauty salon (=0.745), gas station (=0.652) and so on), all of them presenting scaling law with the population size, across 47 US cities. It is possible to note an evident sublinear scaling for all the amenities presented. The data used were collected directly from the references [31,32].

Let us now focus on the infrastructure of the cities. In the present work, the focus is directed to the number of amenities a city has to offer. They are related to the infrastructure sector, and they usually present sublinear behaviour, as shown by figure 2. We built this histogram using 74 kinds of amenities (e.g. bakery (*β*_infra_=0.847), beauty salon (=0.745), gas station (=0.652) and so on) across 47 US cities. The data were collected directly from the references [31,32], but other studies found similar results [9,29]. Although the model we present below relates to the number of amenities in a city, the framework must be valid to other infrastructure sectors of the city.

Suppose *U* to be the total consumption of an individual human need quantity, and then *U*∼*N* [9]. This consumption is supplied by the amenities of the infrastructure sector in consideration. For instance, *U* can be the total consumption of bread in the city, and the amenities that supply this product are the bakeries. The average *per capita* demand of this product is 〈*u*〉=*U*/*N*.

Considering *u*_*i*_ as the consumption of the product (following the last example, the consumption of bread) by a single citizen *i*, we have $U=\sum_{i=1}^{N}u_{i}$. The citizen can acquire this product in *P* amenities distributed around the city, and the choice of what amenity through which he/she will get the product depends on many factors, such as the quality of services, conservation, overcrowding, price, distance, etc. However, we will consider an ideal situation that all the amenities are completely similar, and the only relevant variable for the citizen is the distance. In this way, a rational citizen will choose the amenity that minimizes the transport cost. In other words, we can use the function *f*(*r*_*ik*_), given by equation (2.1), to represent the total supply of the amenity *k* for citizen *i*, and they are separated by the distance *r*_*ik*_. Then the total demand—the provision—of citizen *i* can be computed by

2.12 $$u_{i}=\sum_{k=1}^{P}f(r_{ik}).$$

Here, we assume that the citizen is more influenced, or more supplied in this context, by closest amenities.

In order to compute the number of amenities of a given infrastructure sector, let us to compute first the total provision (of a particular product) of the city. It can be reached by the sum

2.13 $$U\equiv \sum_{i=1}^{N}u_{i}=\sum_{i=1}^{N}\sum_{k=1}^{P}f(r_{ik}),$$

which can also be written as

2.14 $$U=\sum_{k=1}^{P}\left(\sum_{i=1}^{N}f(r_{ik})\right).$$

The sum inside the parenthesis can be computed in a similar way that was done in the previous section, which conducts us to $\sum_{i=1}^{N}f(r_{ik})∼N^{1-\gamma /D_{f}}$, and then (from equation (2.14))

2.15 $$U∼PN^{1-\gamma /D_{f}}.$$

As *U*∼*N*, the above equation yields to

2.16 $$P∼N^{\beta_{\mathrm{infra}}},$$

where

2.17 $$\beta_{\mathrm{infra}}\equiv \frac{\gamma }{D_{f}}$$

is the scaling exponent associated with the infrastructure.

If we are in the *long-range interaction regime*, that is (*γ*/*D*_f_)<1, the number of amenities behaves sublinearly in relation to *N*. Since the empirical data suggest a sublinear behaviour, the theory predicts that the individuals search for gas stations or other infrastructure variables in an interaction of long range. That is, there will be scale economies in infrastructure variables (sublinear behaviour) only if the city interacts as a whole, as a single organism. If the citizens use only the amenities that are in their blocks, it means that the citizens do not use effectively the city, but only a part of it. In this case, we observe a linear relation between the number of amenities (infrastructure) and the population size, which is not consistent with the empirical evidence.

In conclusion, and connecting with what we described in the previous section, when the city interacts as a whole, and not just as a set of isolated parts, then there is improvement of the socio-economic indicators (superlinear behaviour). Consequently, the infrastructure variables show scale economies (sublinear behaviour). Moreover, the bigger the interaction range between citizens and amenities (i.e. the smaller *γ* is), the bigger the improvement of the socio-economic indicators and the lower the infrastructure costs of the city are.

### 2.3. Relation between the scale exponent and the fractality of the city

**Figure 3.**
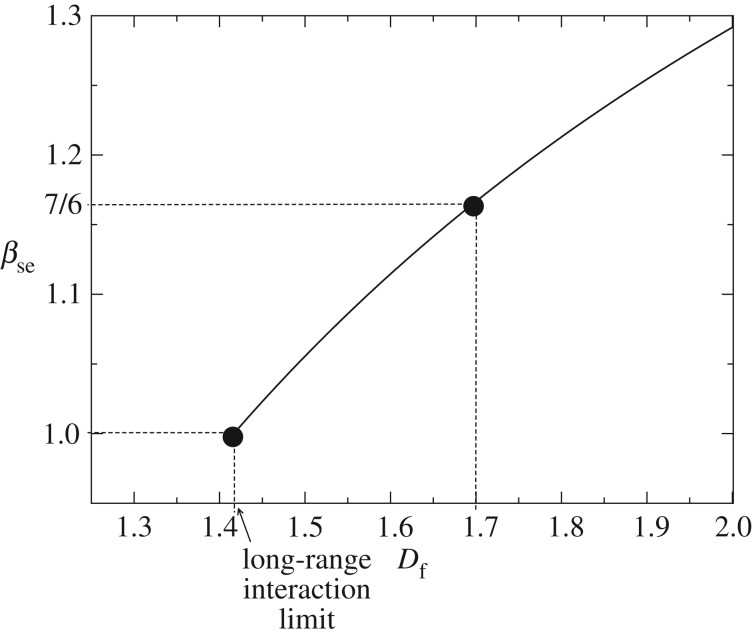
The socio-economic scale exponent as a function of the fractal dimension of the city, according to the equation (2.10), for *γ*=1.41666…. The socio-economic scale exponent is a monotonically increasing function of the fractal dimension of the city. When one considers, by approximation, that the city is a compacted structure, that is *D*_f_=*D*=2, it implies an overestimation of the scale exponent. The extension of the solid line (*D*_f_>*γ*=1.41666…) represents the long-range interaction regime. For *D*_f_<*γ*, that is the short-range interaction regime, the socio-economic variables scale linearly with the population size.

In the long-range interaction regime the scale exponent *β*_se_ is a monotonically increasing function of the fractal dimension of the city, as suggested by equation (2.10) and shown in figure 3. It means that more compact cities (*D*_f_≈2) tend to have bigger scale exponents than fractal cities (*D*_f_<2). That is because, according to our theoretical results, the more compact the city is, the bigger the cultural and social stimuli are. This assertion is in accordance with Bettencourt’s discussion [10]. In his urban mobility-based model, assuming that the population completely fills the area, the scale exponent predicted by his theory is bigger than the one observed experimentally. According to Bettencourt, this is related to the fact that the population does not occupy all the space, but only a fragment of that. The model presented here and consequently the result (2.10), based on social stimulus, also follows the same interpretation.

Given that the three parameters of the result (2.10) are accessible experimentally, the theory can be tested. For instance, if $\beta_{\mathrm{se}}=\frac{7}{6}$ and *D*_f_=1.7, then *γ*=1.41666…, which coincides with the interval of numeric values that the experimental results suggest (between 1 and 1.5). Moreover, this numeric value strengthens the evidence that cities present a long-range interaction regime because (*γ*/*D*_f_)=(1.41666…/1.7)<1.

## 3. Model simulations

**Figure 4.**
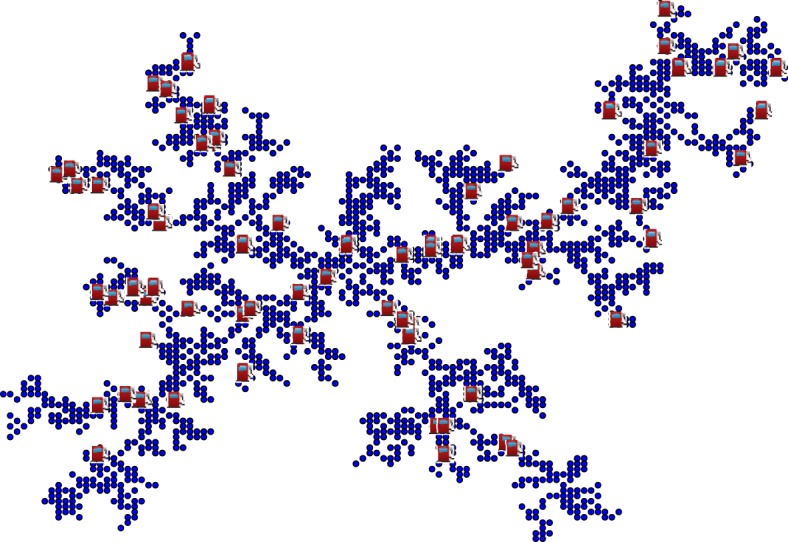
Spatial distribution at equilibrium of a given simulated city. The citizens, *N*=2000 fixed agents represented by the small circles, were distributed according to the DLA algorithm, forming a structure with fractal dimension *D*_f_≈1.7. The amenities, represented by a fuel dispenser, were generated by the rules described in the §3.

In this section, we present computational simulations in order to test the proposed theory. The algorithm used to simulate a virtual city is described as follows. Consider *N* fixed individuals spatially distributed, whose stimulus between them and the total stimulus of the city are computed by equations (2.2) and (2.3), respectively. We will consider two types of spatial configurations in the simulations: compacted and fractal landscape. In the first one, the individuals are fixed and randomly distributed in a square. In this case, the population (compacted) presents dimension *D*_f_=*D*=2. The other type of spatial distribution is fractal and generated by a *diffusion-limited aggregation* (DLA) algorithm [27,33]. One particular configuration generated by this algorithm is the one presented in figure 4 (small circles). In this case, the spatial distribution of the population has dimension *D*_f_≈1.7 [27]. In fact, this artificial structure has been used to model growth of cities due to the numerical similarity between its fractal dimension and the dimension of real cities [24,34]. We will use these two kinds of spatial distribution (compacted and fractal) to see, computationally, how the socio-economic and infrastructure scale exponents behave according to the dimension of the spatial structure formed by the population.

The list below summarizes the algorithm used to simulate a city and to compute the socio-economic variables:

1. First, it generates a city with *N* individuals: using DLA algorithm (*D*_f_=1.7) or distributing the individuals randomly in a square (*D*_f_=2).
2. It computes the distance *r*_*ij*_ between any two individuals.
3. It computes the stimulus of all individuals of the population ({*I*_*i*_}_*i*=1…*N*_), in which $I_{i}=\sum_{j=1}^{N}f(r_{ij})$, and *f*(*r*_*ij*_) is given by equation (2.1). The parameter *γ* is kept fixed throughout the process.
4. Lastly, it computes the total stimulus of the city by $I_{\mathrm{se}}=\sum_{i=1}^{N}I_{i}$.

**Figure 5.**
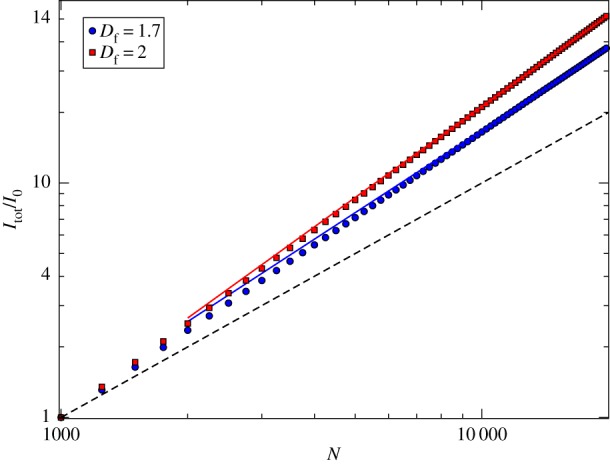
Superlinear behaviour between the total stimulus of the city and the population size, presented by the simulation of the model. The parameters of simulations were: *γ*=1.41666…, *D*_f_=2 (homogeneous distribution) and *D*_f_= 1.7 (DLA algorithm). Points (circles and squares) represent averages over 50 independent samples of numerical simulations, where each simulation is performed keeping *N* fixed. The error bars are smaller than the size of the points. The continuous lines are theoretical predictions (equation (2.10)) where $\beta_{\mathrm{se}}=\frac{7}{6}$ (blue line) and *β*_se_=1.29166 (red line). Dashed line represents the linear scaling.

Figure 5 presents the average of the total stimulus of the two kinds of spatial structure as a function of the population size when *γ*<*D*_f_. It is evident, by the simulation results that the socio-economic exponent follows a superlinear behaviour (*β*_se_>1). That is, the total stimulus of the city is boosted with increases in population size. In relation to the quantitative analysis of the social exponent, there is also a good agreement between the simulation and the analytic prediction given by (2.11). The comparison between the theoretical predictions of the socio-economic exponent and the simulation results is better for a larger population size, due to the minimization of the finite size effects. As was expected, a compact structure presents a greater socio-economic exponent than the fractal one. In other words, the more compact the population is, the more efficient the city will be in the socio-economic aspects.

To simulate the dynamics of the amenities, which represent an infrastructure sector, we used the following algorithm. Given a city generated by the previous algorithm, we have *P*=*P*_0_ amenities (initial condition) that are randomly distributed in the space of the virtual city. Then the provision function of all the individuals of the population is computed via equation (2.12). The average individual provision can be computed by $\bar{u}=(1/N)\sum_{i=1}^{N}u_{i}$. If $\bar{u}<c$ (supply shortages), where *c* is a constant, then one new amenity is introduced in a random position in the city. If $\bar{u}\geq c$, then the less efficient amenity is deleted (bankruptcy) from the city. As the time evolves, the number of amenities converges to an equilibrium (optimal) value. Figure 4 shows a particular city generated using DLA algorithm and the distribution (at equilibrium) of the amenities generated by the algorithm described above.

The algorithm used to simulate the amenities and the variables associated with infrastructure is summarized below:

1. It generates a city with *N* individuals by the same procedure described above.
2. It generates *P*_0_ amenities, each of them is allocated randomly in some point of the city.
3. *P*:=*P*_0_.
4. It computes the distance from any amenity to all the other citizens to obtain {*r*_*ik*_}_*i*=1,…,*N*,*k*=1,…,*P*_.
5. It calculates the supply function of all individuals, that is the set {*u*_*i*_}_*i*=1…*N*_, and the average individual-used provisions, by $u_{i}=\sum_{k=1}^{P}f(r_{ik})$ and $\bar{u}=(1/N)\sum_{i=1}^{N}u_{i}$, respectively.
6. If $\bar{u}>c$, where *c* is a constant, then the amenity that produces less provision to the individuals is deleted. Then do *P*:=*P*−1. Otherwise ($\bar{u}\leq c)$, a new amenity is generated and allocated randomly in some point of the city. Then *P*:=*P*+1.
7. Back to the item 4.
8. The simulation stops when the number of amenities converges.

**Figure 6.**
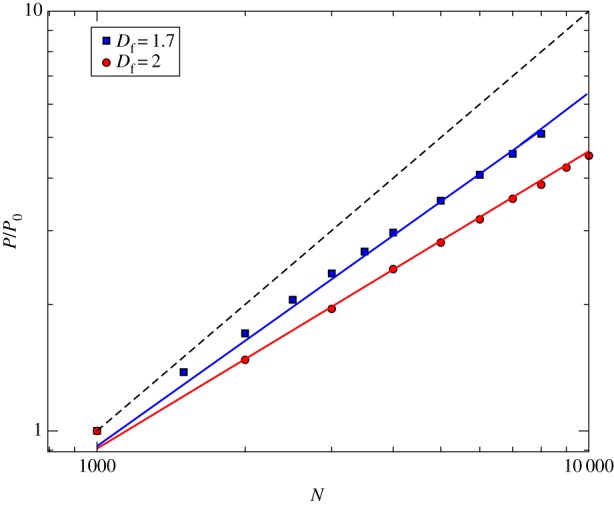
Sublinear behaviour between the number of amenities at equilibrium (normalized by the division by *P*_0_≡*P*(*N*=1000)) and the population size, presented by the simulation of the model. The parameters of simulations are: *γ*=1.41666…, *D*_f_=2 (homogeneous distribution) and *D*_f_=1.7 (DLA algorithm). Points (circles and squares) represent averages over 30 independent samples of numerical simulations, where each simulation is performed keeping *N* fixed. The error bars are smaller than the size of the points. Continuous lines are theoretical predictions (equation (2.16)) where $\beta_{\mathrm{infra}}=\frac{5}{6}$ (blue line) and *β*_infra_=0.708 (red line). Dashed line represents the linear scaling.

Figure 6 shows us that the equilibrium quantity of amenities scales sublinearly with the population size (given *γ*<*D*_f_, i.e. long-range interaction regime). That means we have scale economies, and therefore, greater cities need less amenities *per capita*. These scale economies (sublinear behaviour), according to the model, are a direct consequence of the long-range interaction regime. The opposite situation, that is the short-range interaction regime, must conduct to a linear behaviour, which does not correspond to the empirical evidence.

## 4. Discussions

In our model, the ratio *γ*/*D*_f_ plays an important role. Being *γ*/*D*_f_>1 implies a short-range interaction between the population, that will tend to form isolated groups, and then the city would not behave as an unit. In this situation, as shown by equation (2.9) in this limit, the socio-economic variables will scale linearly with the population size, which is not supported by the empirical data. However, *γ*/*D*_f_<1 implies a long-range interaction between the population and the socio-economic variables will exhibit superlinear behaviour, as shown by equation (2.11), in accordance with empirical evidence.

In the context of this model, because we observe the superlinear behaviour, we expect that the individuals interact in a long-range manner in the cities. So, the definition of what is a city and how to measure its properties must be done with the following requirement in mind: it is not the political borders nor the arbitrary geographical borders that matters. What defines the cities is the geographical areas with interacting population, the so-called *functional cities* [10].

In the following, we discuss the main contributions of the present theory that can shed light into the study of cities.

### 4.1. The explicit dependence of the scaling exponents

One of the main contributions of this work is to obtain an explicit dependence of the scaling exponents *β*, equations (2.10) and (2.17), with ratio *γ*/*D*_f_. As defined in this work, the decay exponent of interaction *γ* and the fractal dimension of the city *D*_f_ are, in principle, both measurable.

As stated by equation (2.10) and shown in figure 3, the scaling exponent associated with the socio-economic activities *β*_se_ is a monotonically increasing function of the fractal dimension of the city *D*_f_. As a consequence, for given *γ*, the denser the city, the greater the socio-economic scaling exponent, more intense the social interaction and so the city development, in accordance with [10]. As stated by equation (2.17), a similar reasoning is valid for the scaling exponent associated with the infrastructure *β*_infra_.

### 4.2. Individual productive capacity

One interesting aspect of the model proposed concerns the *individual productive capacity*, called *G* by Bettencourt [10]. He has shown that this quantity can be expressed as the product of the socio-economic production *per capita* and the infrastructure *per capita*, which reveals to be scale invariant: *G*∼*N*^0^. In the particular case of Bettencourt’s empirical studies, it was shown that these properties of *G* can be observed when we use variables related to area (for instance, road surface area, circumscribing land area, etc.) as the infrastructure variable. In this work, one can show that this property of *G* can also be observed when we use the number of amenities (*per capita*) as the infrastructure variable. That is, according to the results given by equations (2.10) and (2.17),

4.1 $$G=\left(\frac{I_{\mathrm{se}}}{N}\right)\cdot \left(\frac{P}{N}\right)∼N^{2-\gamma /D_{f}-1}N^{\gamma /D_{f}-1}=N^{0},$$

which means *G* is a constant in relation to *N* (in the absence of a scaling law), regardless of the numeric values of *D*_f_ or *γ*. Thus, the result of the current work is compatible with the fact that *G*, being related to individual effort and thus limited to physical constraints, should not depend on the size of the city.

### 4.3. Relation between socio-economic and the infrastructure scaling exponents

Another important feature that emerges from the model is the relation between the socio-economic and infrastructure scaling exponents. For instance, the sum of these exponents,

4.2 $$\beta_{\mathrm{se}}+\beta_{\mathrm{infra}}=\left(2-\frac{\gamma }{D_{f}}\right)+\left(\frac{\gamma }{D_{f}}\right)=2$$

is a number (=2), which does not depend on the parameters of the model (*γ* or *D*_f_). Moreover, this result corroborates the same relation found in [7] and it is in accordance with the empirical data, presented in table 1.

### 4.4. Different decay exponents

In our model, we are supposing that the decay exponent *γ* is the same for both socio-economic and infrastructure variables. However, the way citizens behave in relation to them can be different. Therefore, if we build a *γ* for each variable as: *β*_infra_=*γ*_infra_/*D*_f_ and *β*_se_=2−*γ*_se_/*D*_f_, the sum of the scaling exponents will be

4.3 $$\beta_{\mathrm{se}}+\beta_{\mathrm{infra}}=2+\frac{\gamma_{\mathrm{infra}}-\gamma_{\mathrm{se}}}{D_{f}}.$$

Note that this sum can be below or above 2. Empirically, as we showed in table 1, the sum varies around 2. There are two alternative explanations for this: (i) this fluctuation is due to some measurement error or (ii) it is due to the difference between *γ*_se_ and *γ*_infra_. More empirical evidence and, consequently, a more detailed study are necessary to clarify this issue.

### 4.5. Different urban variables with different scaling exponents

Now, we consider the possibility that different infrastructure sectors present different scaling exponents. For instance, let’s take two different kinds of amenities in the city: bakeries and gas stations. According to empirical data presented by [31,32], *β*_*bakery*_=0.847, while *β*_*gasstation*_=0.652 in the USA. The model sheds some light on the difference between these values. If *D*_f_ is the same for both, the model says that if *β*_*bakery*_>*β*_*gasstation*_, then *γ*_*bakery*_>*γ*_*gasstation*_, which means that bakeries are more restricted, that is, they have a smaller range of interaction than gas stations. That makes sense, because people usually go to bakeries that are in the neighbourhood. However, it is more common for people to fuel their cars further away from their homes. The same idea can be applied in the context of the socio-economic variables.

### 4.6. The fluctuations in real data

There is one more interesting possibility allowed by the approach presented in this work: it can also give some insights about the fluctuations of the urban variables that are observed in empirical allometries. Empirical data from recent works [35,36] suggest that the residuals surrounding the scaling laws are log-normally distributed. The present model can explain such properties as a consequence of fluctuation of the interaction range of the citizen, given by particularities of the cities.

To understand that, consider the hypothesis that the exponent decay *γ* is Gaussian distributed in an ensemble of cities of the same population size. This implies that the scaling exponent *β* will be also normally distributed, since *β*∝*γ*, which is consistent with the empirical data presented in figure 2. From the allometric equation *Y* ∝*N*^*β*^, being *β* normally distributed and log⁡Y∝βlog⁡N, then log⁡Y will be also normally distributed. Then the urban indicator *Y* , which can represent both a socio-economic *I*_se_ or an infrastructure *P* variable, must be lognormally distributed, in the same way that it is observed in empirical data. In [35], the authors consider Gaussian noise in the multiplicative constant of the scaling equation to explain the lognormal distribution observed in real data. While they assumed *β* fixed without noise, we consider *β* is noise.

In conclusion, the model proposed suggests that the fluctuation in the interaction range of the individuals conducts to the lognormal distribution of the allometric metrics, in accordance with the empirical facts. In other words, fluctuations in urban variables would be due to the diversity and peculiarities of each city in promoting the spatial integration of individuals.

### 4.7. Practical applications for urban development

Based on the previous considerations, we now address the role of the parameters *γ* and *D*_f_ in practical applications. In general, it is usual to say that the development of a city for good is associated with the increase of the socio-economic scaling exponents, equation (2.10), and the decrease of the infrastructure scaling exponents, equation (2.17). For both exponents, the smaller the ratio *γ*/*D*_f_, the better the city development.

Creating public policies able to decrease the ratio *γ*/*D*_f_ should be the goal of cities’ administration. This could be done (i) by increasing the density of the cities, or what is the same, the fractal dimension *D*_f_; (ii) by decreasing the decay exponent *γ*, which means increasing the interaction range between the citizens, by creating public policies capable of promoting distant individuals to interact by decreasing the cost of transportation, or improving and diversifying urban mobility, for instance; and (iii) by a combination of both, since those variables might be dependent or correlated one to the other.

However, these strategies might be bounded by correlations between what is generally seen as prosperity variables (such as GDP, income, employment, etc.) and what is generally seen as disadvantage variables (such as criminality and *CO*_2_ emission levels). While the increase of the former variables are welcome, the increase of the latter are not. We need first to know to what extent those variables are independent or correlated before discussing specific policies. Empirical evidence regarding the values of *γ* for the different urban variables and their correlations is still missing and needs to be further studied before discussing specific policies.

## 5. Conclusion

This work sheds light on the understanding of scaling laws which emerge in cities. We argued that the cities’ scaling laws observed in empirical data can be explained considering distance-dependent interactions at individual level. We developed a microscopic model approach, based on pair interaction of individuals with distance, characterized by a decay exponent *γ* that accounts for the superlinear and sublinear scaling, respectively, of the socio-economic and infrastructure variables. To obtain scaling exponents compatible with empirical data, the long-range interaction must be prevalent. We built an agent-based model to test the hypotheses behind the presented assumptions. The main idea is that the model can be built without taking some details into consideration. The scaling laws obtained from simulations were compatible with empirical data.

The proposed model presents an explicit relation for the scaling exponents *β* in terms of the decay exponent *γ*, and the fractal dimension *D*_f_ of the city. The model predicts that the sum of the scaling exponents of socio-economic and infrastructure variables are 2, as observed in empirical data, independent of the parameters of the model. The scaling exponents *β* can be different for different socio-economic and infrastructure variables, and we addressed how public policies could take advantage of these properties to improve cities’ development, minimizing negative effects.

The model also presents an alternative interpretation for the lognormal distribution of the scaling variables. It proposes that the fluctuation in the interaction range of the individuals conducts to the log-normal distribution of the allometric metrics, in accordance with the empirical facts. In other words, fluctuations in urban variables would be due to the diversity and peculiarities of each city in promoting the spatial integration of individuals.

This framework elegantly allows researchers to make microscopic measurements of individual interactions and to integrate the outcome with the city geometrical properties to estimate the underlying scaling exponents. This study relies on the fact that cities have to be seen as a unit as their emergent scaling laws depend on the fact that cities work as integrated systems. Further research in this line is warranted.

## Data accessibility

All data used are publicly available and properly cited.
